# Ossification of caroticoclinoid ligament and its clinical importance in skull-based surgery

**DOI:** 10.1590/S1516-31802007000600009

**Published:** 2007-11-01

**Authors:** Srijit Das, Rajesh Suri, Vijay Kapur

**Affiliations:** Department of Anatomy, Universiti Kebangsaan Malaysia, Kuala Lumpur, Malaysia

**Keywords:** Skull, Sphenoid bone, Skull, Abnormalities, Anatomy, Cráneo, Hueso esfenoides, Crânio, Anomalías, Anatomía

## Abstract

**CONTEXT:**

The medial end of the posterior border of the sphenoid bone presents the anterior clinoid process (ACP), which is usually accessed for operations involving the clinoid space and the cavernous sinus. The ACP is often connected to the middle clinoid process (MCP) by a ligament known as the caroticoclinoid ligament (CCL), which may be ossified, forming the caroticoclinoid foramen (CCF). Variations in the ACP other than ossification are rare. The ossified CCL may have compressive effects on the internal carotid artery. Thus, anatomical and radiological know­ledge of the ACP and the clinoid space is also important when operating on the internal carotid artery. Excision of the ACP may be required for many skull-based surgical procedures, and the presence of any anomalies such as ossified CCL may pose a problem for neurosurgeons.

**CASE REPORT:**

We observed the presence of ossified CCL in a skull bone. A detailed radiological study of the CCL and the CCF was conducted. Morphometric measurements were recorded and photographs were taken. The ACP was connected to the MCP and was converted into a CCF. Considering the fact that standard anatomy textbooks do not provide morphological descriptions and radiological evaluations of the CCL, the present study may be important for neurosurgeons operating in the region of the ACP.

## RESUMEN

**CONTEXTO Y OBJETIVO:**

El extremo intermedio de la frontera posterior del hueso del sphenoid presenta el proceso anterior del clinoid (ACP), que está alcanzado generalmente para las operaciones que implican el espacio clinoidal y el sino cavernoso. El ACP es conectado a menudo con el proceso medio del clinoid (MCP) por un ligamento conocido como el ligamento del carotico-clinoid (CCL), que puede ser osificado, formando el agujero del clinoid del carotico (CCF). Las variaciones del ACP con excepción de la osificación, son raras. El CCL osificado puede tener efectos compresivos en la arteria carótida interna. El conocimiento así anatómico y radiológico del ACP y del espacio clinoidal es también importante mientras que funciona en la arteria carótida interna. La supresión del ACP se puede requerir para muchos cirugía basada cráneo y la presencia de cualquier anomalía tal como CCL osificado, puede plantear un problema para el neurocirujano.

**INFORME DEL CASO:**

Observamos la presencia de CCL osificado en un hueso del cráneo. Un estudio radiológico detallado del CCL y del CCF fue conducido. Las medidas morphometric fueron registradas y las fotografías fueron tomadas. El ACP fue conectado con el MCP y convertido en un CCF. En vista del hecho, los libros de textos de esa anatomía del estándar no proporcionan la descripción morfológica y la evaluación radiológica de CCL, el actual estudio puede ser importante para los neurocirujanos que funcionan en la región del ACP.

## INTRODUCTION

The medial end of the lesser wing of the sphenoid bone forms the anterior clinoid process (ACP).^1^ The ACP provides attachment to the free margin of the tentorium cerebelli and is grooved medially by the internal carotid artery.^1^ The ACP is joined to the middle clinoid process (MCP) by the caroticoclinoid ligament (CCL), which is sometimes ossified. A dural fold extending between the anterior and middle clinoid processes or ossification of the CCL may result in the formation of the caroticoclinoid foramen (CCF).^1^

In neurosurgical operations, the ACP is usually accessed in order to gain entry into the clinoid space.^2^ After the internal carotid artery leaves the cavernous sinus, it is related medially to the ACP. The presence of an ossified CCL may form a potential site for compression of the internal carotid artery. Abnormal variations in the ACP may pose a risk while it is being removed in regional surgical procedures.^2^

Knowledge about the ossification of the CCL may be immensely beneficial for skull surgeons. Considering the fact that anatomy textbooks do not provide a detailed description of the anatomoradiological characteristics of the CCL or CCF, the present study may prove especially relevant to neurosurgeons and radiologists in day-to-day clinical practice.

## CASE REPORT

**Figure 1:**
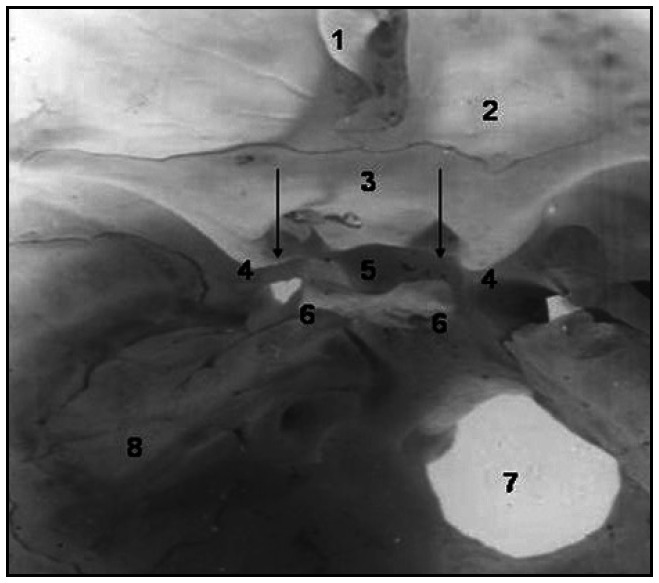
Photograph of interior of skull showing:1. Frontal crest; 2. Orbital part of frontal bone; 3. Jugum sphenoidale; 4. Anterior clinoid process; 5. Pituitary fossa; 6. Posterior clinoid process; 7. Foramen magnum; 8. Petrous part of temporal bone. The ossified caroticoclinoid ligament extending between the anterior and middle clinoid processes is marked by vertical arrows.

**Figure 2:**
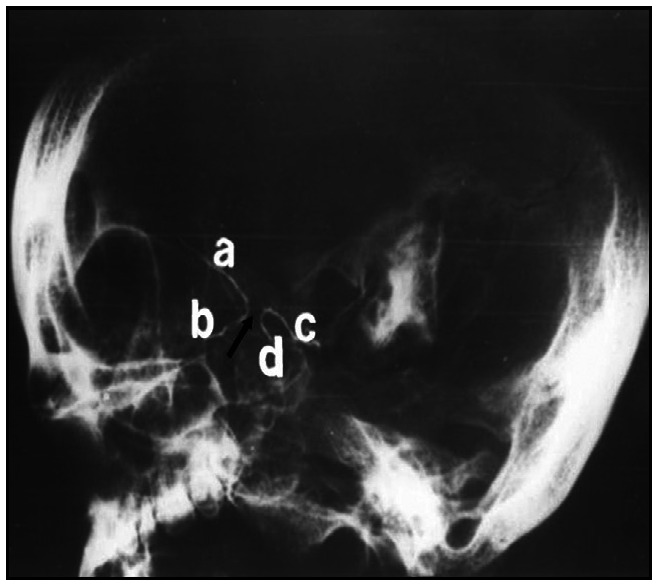
X ray photograph of skull (lateral view), with wires inside optic canal and caroticoclinoid foramen. a. Upper end of bent wire inside optic canal; b. Lower end of bent wire inside optic canal; c. Posterior end of bent wire inside caroticoclinoid foramen; d. Anterior end of bent wire inside caroticoclinoid foramen. The ossified caroticoclinoid ligament is shown with an arrow.

The skull bones kept in the Department of Anatomy are prepared by means of initial washing, followed by autoclaving and treatment with ethylene oxide, and are finally freeze-dried. During routine osteology teaching for undergraduate medical students, we observed an anomalous CCL in a skull bone. Anomalous ossification of the CCL was noted and the specimen was photographed (Figure 1). Appropriate morphometric measurements were recorded and a proper radiological evaluation was also conducted (Figure 2).

The ossified CCL (shown by vertical arrows in Figure 1) was found to extend between the ACP (marked as "4" in Figure 1) and the MCP (marked with an vertical arrows in Figure 1), in the bone specimen. The ossified CCL was found bilaterally, on both sides of the skull. The anterior clinoid processes on the two sides were separated by a distance of 2.1 cm. The anterior and posterior clinoid processes were separated by a distance of 0.7 cm and 0.6 m on the right and left sides, respectively. The sulcus chiasmaticus was situated at a distance of 0.5 cm behind the jugum sphenoidale. The right posterior clinoid process was found to be more prominent (marked as "6" in Figure 1) than on the left side. The ossified CCL measured approximately 1.5 cm on each side. The CCF was prominently formed as a result of the presence of the ossified CCL. The maximum transversal dimension between the optic canals on each side was 1.5 cm.

## DISCUSSION

The ACP forms the attachment site for the free anterior margin of the tentorium cerebelli, whereas the MCP provides the attachment for the diaphragma sellae.^1^ The parts of the sphenoid bone that are usually reported as capable of ossification are the pterygospinous and interclinoid processes.^1^ As described in conventional textbooks of anatomy, the ACP may be joined to the MCP by a ligament or dural fold.^1^ The bony bridge joining the ACP and MCP converts the distal end of the carotid sulcus into an ostium known as the CCL.^2^

To the best of our knowledge, no single osteological study has ever been supplemented with additional radiological findings of a CCL, and the present case is a humble attempt to highlight this finding. A skiagram was taken using separate wires inserted into two different openings, i.e. the optic canal and CCF, for easy differentiation (Figure 2).

The internal carotid artery is present in the medial groove of the ACP and it may be compressed by the ossified CCL, giving rise to vascular complications. The presence of an ossified CCL is likely to cause compression and straightening of the internal carotid artery.^3^ In the present case, the clinical history of the patient was not available to corroborate this observation.

The internal carotid artery is conventionally divided into six segments and the clinoid segment of the artery is located between the proximal and distal dural rings.^4^ In any surgical operation involving exposure of the clinoid segment of the internal carotid artery, excision of the anterior clinoid process is mandatory. Even to expose the cavernous sinus superiorly and to manage paraclinoid aneurysm, the ACP has to be removed.^2,5^ The clustering of the neurovascular structures in the vicinity of the ACP renders the surgery more risky.^2^ Prior anatomical knowledge is essential for identifying any inadvertent injury to the internal carotid artery.

Research studies have also reported the fact that an ossified CCL makes the removal of the ACP more difficult, especially in the presence of any aneurysm.^2^ Drilling of the ACP, when required, may cause inadvertent injury to the internal carotid artery and optic nerve. There have been previous reports of CCF occurrence in dried human skulls.^6^ That particular anatomical study laid less emphasis on the radiological aspects of the ossified CCL.^6^ In comparison to earlier anatomical studies, the present study has not only described the morphological and clinical characteristics of an ossified CCL, but also displayed the radiological features of an ossified CCL, which may be beneficial for radiologists.

Another important clinical characteristic is the pneumatization of the ACP, which has to be evaluated pre-operatively, in order to avoid serious complications like pneumocephalus and rhinorrhea.^7^ For any surgery involving the ACP, preoperative imaging may be advised, to keep such anomalies in view. Interestingly, 60% of ACP cases are pierced by narrow venous canals arising from the anterior cavernous sinus and traversing through the clinoid space. These are considered to be a potential source of bleeding during removal of the ACP.^8^ It must also be remembered that the extraocular nerves traverse to the superior orbital fissure inferolaterally to the ACP, and it is essential for surgeons to adopt a careful approach when operating on the ACP.^9^

## CONCLUSION

If an ossified CCL is present, it is likely to cause compression of the internal carotid artery. A detailed anatomical report of such an anomaly was presented in the present case. Radiological studies on the CCF and its differentiation from the optic canal may be clinically important for radiologists. Anatomical knowledge about ossification of the CCL may be useful in cases of surgery involving removal of the ACP, for which additional risk is involved.
